# A network analysis of research productivity by country, discipline, and wealth

**DOI:** 10.1371/journal.pone.0232458

**Published:** 2020-05-13

**Authors:** Klaus Jaffe, Enrique ter Horst, Laura H. Gunn, Juan Diego Zambrano, German Molina

**Affiliations:** 1 Universidad Simon Bolivar, Caracas, Venezuela; 2 Facultad de Administracion, Universidad de los Andes, Bogota, Colombia; 3 Department of Public Health Sciences, University of North Carolina at Charlotte, Charlotte, North Carolina, United States of America; 4 School of Public Health, Imperial College London, London, United Kingdom; 5 Idalion Capital Group, London, United Kingdom; Shandong University of Science and Technology, CHINA

## Abstract

**Introduction:**

Research productivity has been linked to a country’s intellectual and economic wealth. Further analysis is needed to assess the association between the distribution of research across disciplines and the economic status of countries.

**Methods:**

By using 55 years of data, spanning 1962 to 2017, of Elsevier publications across a large set of research disciplines and countries globally, this manuscript explores the relationship and evolution of relative research productivity across different disciplines through a network analysis. It also explores the associations of those with economic productivity categories, as measured by the World Bank economic classification. Additional analysis of discipline similarities is possible by exploring the cross-country evolution of those disciplines.

**Results:**

Results show similarities in the relative importance of research disciplines among most high-income countries, with larger idiosyncrasies appearing among the remaining countries. This group of high-income countries shows similarities in the dynamics of the relative distribution of research productivity over time, forming a stable research productivity cluster. Lower income countries form smaller, more independent and evolving clusters, and differ significantly from each other and from higher income countries in the relative importance of their research emphases. Country-based similarities in research productivity profiles also appear to be influenced by geographical proximity.

**Conclusions:**

This new form of analyses of research productivity, and its relation to economic status, reveals novel insights to the dynamics of the economic and research structure of countries. This allows for a deeper understanding of the role a country’s research structure may play in shaping its economy, and also identification of benchmark resource allocations across disciplines for developing countries.

## Introduction

Research and economic progress are intertwined concepts. This relationship has a long history in the literature [1–4]. However, the nature of the inner workings of this relationship in the modern world is still controversial regarding the relative importance of different fields of research in the economic development of nations [5–9]. The traditional view is that research advancement is required for technological expansion, which affects the productive apparatus of a country explaining a high correlation between research and technological advances [10,11]. This, in turn, explains why research advancement and the wealth of countries are closely linked [12]. A significant contribution to the debate was the modeling of the network structure and connectedness from a purely economic standpoint [13]. However, economic diversification is not the only relevant factor, and not all knowledge is equal. Different types of knowledge might be relevant for different techno-economic expansions. Previous work [9,14] showed that research productivity of middle income countries correlates stronger with present and future wealth than indices reflecting its financial, social, economic, or technological sophistication.

Many approaches to the assessment of relationships between research productivity and economic advancement do not disaggregate this linkage by discipline, by country, or both, with narrow but deep analysis of particular linkages, yet lacking a global, comprehensive view [15–20].

This manuscript builds and expands on previous work [9,21], which identified the contribution (or association) of the relative productivity across different disciplines in predicting the future economic growth of a country, showing that wealthy and poor countries differ in the relative proportion of their research output in the different disciplines. Countries with higher relative productivity in basic sciences, such as physics and chemistry, had the highest economic growth in the following five years compared to countries with a higher relative productivity in applied sciences such as medicine and pharmacy [9]. The results of this prior study also indicated that middle income countries that focused their research efforts in selected disciplines grew slower than countries which invested in general basic sciences. This study analyzed correlations between successive time periods, suggesting probable (Granger) causation among certain variables, but not demonstrating it. Their approach was also limited geographically, temporally, and in terms of the disaggregation across research disciplines.

By using a more extensive information set and building on a novel network approach in the field of comparative research, this manuscript provides a deeper insight into aspects of the relationships among disciplines, and between those disciplines and countries’ economic development. Note, however, that those associations can be linked to, or driven by, other latent factors not described in this analysis.

This manuscript introduces a method for assessing relationships that relies on the extraction of the relative research backbone network [22]. The method is robust to both total research productivity and country size, providing a normalized approach to research productivity analysis and its relationship to a country’s economic status. This approach explores the structure of a country’s research ecosystem, characterized by the relative productivity of the diverse research disciplines, across a wide set of countries of different wealth status. Relationships are assessed across time, discipline, and countries. Data-driven constructs (clusters) are extracted, which group countries with similar research productivity profiles (irrespectively of whether the countries’ researchers collaborate with each other), as well as group disciplines with similar profiles among countries. This novel approach provides an insight to the evolution of discipline clusters as well as how country clusters relate to the underlying economic income status of the country. The objectives of this manuscript are: (1) Explore the similarities and clusters in research profiles between different countries; (2) Assess whether those similarities are associated with country income status; (3) Explore the relationships among disciplines based on similarities between them in country-based research profiles; and (4) Describe the evolution over time of country-based research profiles and clusters where those countries belong, given their different political and economic paths.

## Methods

### Study inclusion and exclusion criteria

Data for the number of peer-reviewed research publications between 1963 and 2017 across countries was gathered using Elsevier Developer Application Programming Interface (API) with an HTTP connection to the Scopus database. The data was downloaded using an Elsevier API (dev.elsevier.com) together with code written in Python using the Python library script found at github.com/ElsevierDev/elsapy.

The classification for each publication in its respective discipline was grouped according to the subjects defined by Elsevier's SciVerse Scopus All Science Journals Classification (ASJC), which is based on the article abstract, scope, and similar contents that the journal publishes. Each of these ASJC codes was used to filter the number of publications in the queries to the API.

Only articles and conference papers were included in the dataset. Reviews, book chapters, viewpoint articles, and letters were excluded from the analysis. All of the listed journals in the Scopus database were selected. However, journals that stopped publishing during the sample periods were not included, since full information was not available.

Countries for which the data was incomplete or unreliable for any year or discipline at the time of data extraction, such as Brazil or Argentina, were excluded from the study. Countries which underwent significant transformations, such as Russia or Germany were also excluded from the study to avoid artificial sharp changes at the time of their geographical or geopolitical redefinitions (integration or disintegration). This led to a study universe comprising 62 countries.

Regarding the country classification of wealth, World Bank classifications [23] were used to map countries to low, low-medium, high-medium, and high income for the purpose of sub-group analyses. The only political entity explicitly excluded was Taiwan, since the World Bank gross domestic product (GDP) report [23], used in this manuscript for income classification, did not include Taiwan in their database. Tables 1 and 2 provide lists of research disciplines and countries, respectively, included in this study. While the scope is not comprehensive, it includes all countries with full history across all time periods.

**Table 1:** Abbreviations for the subject area classifications included in the analysis, as described in Elsevier’s All Science Journal Classification (ASJC) scheme.

Full name	Code
Agricultural and Biological Sciences	AGRI
Arts and Humanities	ARTS
Biochemistry, Genetics, and Molecular Biology	BIOC
Business, Management, and Accounting	BUSI
Chemical Engineering	CENG
Chemistry	CHEM
Computer Science	COMP
Decision Sciences	DECI
Dentistry	DENT
Earth and Planetary Sciences	EART
Economics, Econometrics, and Finance	ECON
Energy	ENER
Engineering	ENGI
Environmental Science	ENVI
Health Professions	HEAL
Immunology and Microbiology	IMMU
Material Science	MATE
Mathematics	MATH
Medicine	MEDI
Multidisciplinary	MULT
Neuroscience	NEUR
Nursing	NURS
Pharmacology, Toxicology, and Pharmaceutics	PHAR
Physics and Astronomy	PHYS
Psychology	PSYC
Social Sciences	SOCI
Veterinary	VETE

**Table 2:** Universe of countries meeting the study inclusion criteria and included in the analysis, as well as manuscripts per country.

Country	# of Publications
Algeria	104,865
Australia	2,474,831
Austria	703,534
Bahamas	1,058
Bangladesh	72,937
Belgium	1,005,508
Botswana	11,377
Burkina Faso	10,353
Canada	3,448,259
China	9,168,307
Colombia	146,723
Congo	8,747
Costa Rica	44,147
Cote d’Ivoire	11,738
Denmark	734,280
Dominican Republic	2,622
Ecuador	24,149
Fiji	6,138
Finland	635,477
France	4,219,564
Ghana	30,500
Greece	536,776
Honduras	2,206
Hong Kong	483,545
Iceland	35,652
India	2,915,920
Israel	800,533
Italy	3,216,051
Jamaica	14,432
Japan	5,965,525
Kenya	62,236
Luxembourg	30,742
Madagascar	6,770
Malaysia	450,197
Mexico	525,058
Morocco	93,935
Netherlands	1,876,572
New Zealand	442,962
Nigeria	160,319
North Korea	1,876
Norway	561,384
Pakistan	211,105
Papua New Guinea	7,180
Philippines	51,321
Rwanda	4,445
Senegal	17,242
Sierra Leone	2,045
Singapore	497,132
South Africa	483,413
Spain	2,314,197
Sri Lanka	32,286
Sudan	17,954
Sweden	1,313,660
Thailand	275,024
Trinidad & Tobago	12,002
Turkey	852,916
Uganda	26,693
United Kingdom	7,058,367
United States	25,912,014
Uruguay	31,703
Zambia	10,935
Zimbabwe	19,493

Research products are assigned to each of the host countries of the contributing authors. By assigning in full each article to each researcher’s host country, the research contribution and productivity per country is more fairly reflected. Assigning a manuscript to a single country (e.g., that of the first author) could penalize work by researchers in lower-productivity countries who may disproportionately rely on joint research with colleagues in higher-productivity countries. Splitting the weight of each manuscript (evenly or otherwise) among countries of the co-authors would also be inadequately reflecting the contributions of each country or unevenly assessing the research efforts per discipline. For example, medical manuscripts oftentimes have a dozen or more co-authors in multiple countries, while mathematical articles tend to have much fewer (oftentimes in a single country). A split of the weight of articles per author/host country could unfairly over-estimate the research productivity in the mathematical discipline relative to the medical discipline. The raw data used in this manuscript is available at https://doi.org/10.3886/E118746V1.

### Analysis

The number of publications per country and discipline over the period of analysis is likely upward trending and non-stationary, with growing numbers of publications per year for most countries and disciplines. This is likely to be especially prevalent for incipient research disciplines, growing with increased maturity of the disciplines and penetration within higher education institutions (e.g., computer sciences), as well as country’s population and wealth [24]. To remove the effect on the analysis of this uptrend, we define *P*_*t*,*c*,*f*_ as the share of publications for country *c* relating to research field *f* in year *t*, where publications are normalized so that **1**^*T*^***P***_*t*,⦁,*f*_ = 1 for each vector of country publication shares ***P***_*t*,⦁,*f*_ (where ⦁ denotes all possible categories of a given index–in this example, all possible countries).

Country shares of publications for each research field evolve over time, not only depending on the absolute level of research produced by the country, but also in relative terms to the rest of the world’s research in that discipline. If two research fields, *f*_1_ and *f*_2_, are similar, they are expected to define similar vectors of country shares $P_{t,⦁,f_{1}}\approx P_{t,⦁,f_{2}}$, as a reflection that research productivity in one discipline does not occur in isolation from research in the other.

Let *d*(**Ω**, Ϛ, *f*_1_, *f*_2_) be a distance measure between research fields *f*_1_ and *f*_2_ over a given period **Ω** (which can be single or multi-year) for a subset of countries Ϛ. The distance function *d* is defined in Euclidean Distance Matrix form [25,26] and is cumulative over the period **Ω**, with non-diagonal elements:

$$d(\Omega ,Ϛ,f_{1},f_{2})=\sum_{t\subset \Omega }\|P_{t,Ϛ,f_{1}}-P_{t,Ϛ,f_{2}}{\|}^{2}.$$

Research field *f*_1_ is closer to field *f*_2_ than to field *f*_3_ during period **Ω** for the subset of countries Ϛ when *d*(**Ω**, Ϛ, *f*_1_, *f*_2_) < *d*(**Ω**, Ϛ, *f*_1_, *f*_3_). Therefore, a weight function of linkages between research fields can be defined as *w*(**Ω**, Ϛ, *f*_1_, *f*_2_) = *d*(**Ω**, Ϛ, *f*_1_, *f*_2_)^−1^. An undirected graph representing the relationships between research fields for a subset of countries Ϛ, during period **Ω** is composed of pairs of fields *f*_1_ and *f*_2_ (vertices in the graph) connected through edges with weights *w*(**Ω**, Ϛ, *f*_1_, *f*_2_). This is performed using the *graph*.*adjacency* routine within the *igraph* package in R.

Since all pairs of vertices are connected through edges with non-zero weight, all graphs are effectively complete. However, most edges may have low (relative) weights, indicating lower levels of relative connectedness between research fields. In order to extract the backbone structure (set of relationships that remain after a statistical network reduction approach is implemented), we follow [22] and apply their proposed disparity filter using the *disparityfilter* package in R. This approach extracts the network with edges that are statistically significantly (α = 0.05) away from the null model (complete network), without penalizing research fields that may be proportionally smaller or less connected. This approach produces significantly more sparse graphs that represent only statistically significant edges between connected vertices. Note that the value of the significance level (α = 0.05) is pre-specified ahead of the analysis to avoid data snooping. Higher values of α provide more dense networks, while smaller values will result in more sparse networks. As with most statistical approaches, the value of α = 0.05 is both conventional and arbitrary.

Upon constructing the backbone network, a fast greedy algorithm [27] is used to define the optimal community structure of the graph using the *cluster_fast_greedy* algorithm also within the igraph package in R. These communities allow for dense groups of vertices that share commonalities to be grouped together, forming natural clusters of research fields based on their relationships with other fields. This is represented graphically by splitting the edges into intra-cluster edges (black) and inter-cluster edges (red). Intuitively, nodes connected through black edges belong to a cluster that shares similar features, while those linked through a red edge represent vertices that, while belonging to different clusters, are still similar. For the purpose of graph simplicity, only vertices with at least one connecting edge upon performing the disparity filter are included as part of the resulting graph.

Graphical representations of countries based on their similarities across research disciplines are also possible through the adaptation of the distance function to measure the distance between pairs of countries across research discipline productivity shares. This denotes countries that are similar in both absolute and relative research productivity across all fields (though subsets of fields are also possible). For any given pair of countries *c*_1_ and *c*_2_, the distance is defined across fields as follows:

$$d*(\Omega ,c_{1},c_{2})=\sum_{t\subset \Omega }\|P_{t,c_{1},⦁}-P_{t,c_{2},⦁}{\|}^{2},$$

and the corresponding weights w* are defined as the inverses of the non-diagonal components of the new Euclidean Distance Matrix. While other distance measures were possible (e.g., Manhattan, Cosine, etc.), we chose the Euclidean distance for interpretability.

Finally, country research productivity shares are expected to be largely driven by country size. A measure that further normalizes the research productivity shares (scaling them to add to one across disciplines for each country) is needed for country size-adjusted comparisons of inter-disciplinary shares across countries when using the Euclidean distance. This can be achieved by normalizing the research productivity shares *P* over all possible countries *c*. This leads to the final, normalized version of the networks, introduced in this study, where countries of differing sizes are compared.

Note that, while the study is cross-sectional for each 5-year period defining each of the aforementioned probability vectors, there is an inter-temporal component in the analysis of the differences of those analyses over time.

## Results

**Fig 1:**
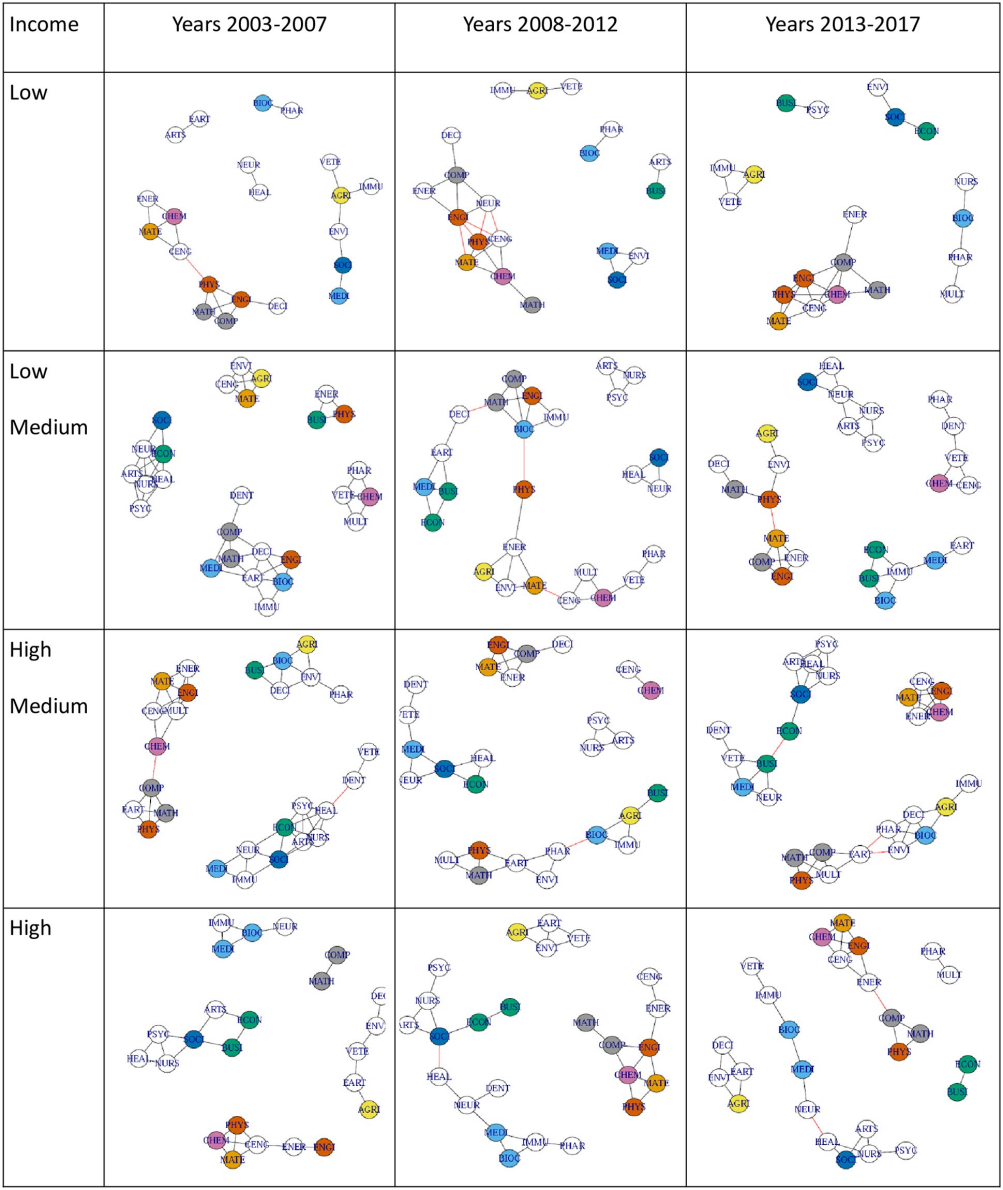
Discipline networks by income cluster (rows) and lustra five-year period (columns). Edges represent closeness in research productivity shares across countries. Vertices colors aid visualization of relationships: (1) light blue for Biochemistry and Medicine; (2) green for Economics and Business; (3) grey for Computer Sciences and Mathematics; (4) dark orange for Engineering and Physics; (5) light orange for Materials; (6) yellow for Agriculture; (7) pink for Chemistry; (8) dark blue for Sociology; and (9) no color for the remainder (see Table 1 for the full scope of the aforementioned disciplines).

**Fig 2:**
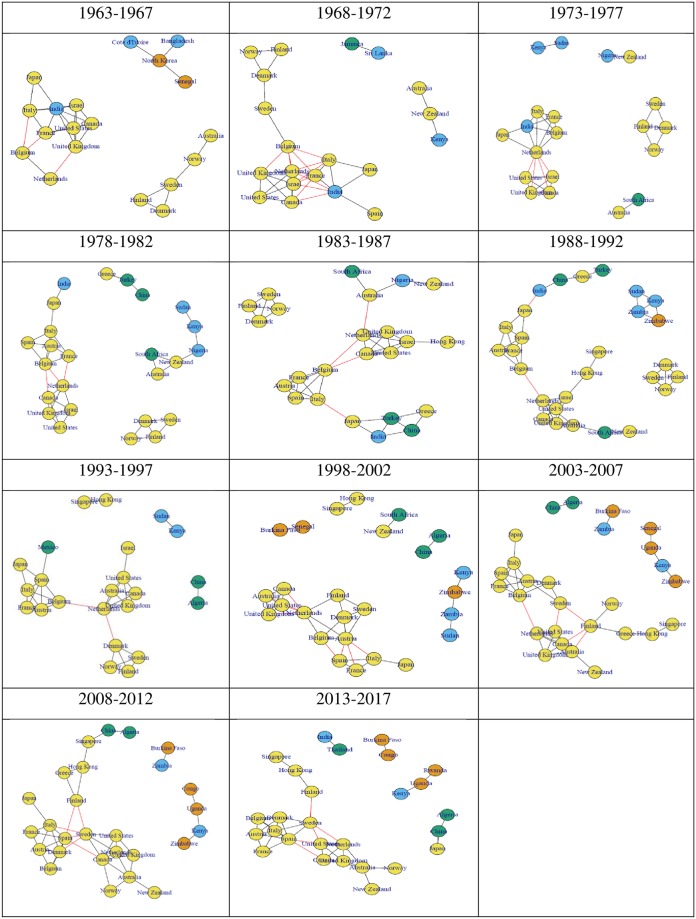
Country networks by five-year periods lustra with edges representing closeness in research productivity shares across disciplines and vertices colors representing World Bank economic classifications (from left to right, e.g., 1963 to 1967 (top left), …, 1973 to 1977 (top right), …, 2013 to 2017 (bottom middle)). Color-coding was based on the country classification by income produced by the World Bank [23]: (1) yellow for high income countries; (2) green for medium-high income countries; (3) blue for medium-low income countries; and (4) orange for low income countries.

Results are represented graphically in Figs 1 and 2. The graphs in Figs 1 and 2 represent relationships (networks) between different research disciplines by income across countries (Fig 1), as well as networks between different countries across disciplines (Fig 2), in both cases over multiple 5-year periods. The distributions of log-weights and log-distances corresponding to most graphs within Figs 1 and 2 are relatively uniform.

In Fig 1, the color associated with each research discipline was selected (subjectively) to visually enhance key disciplines and expected relationships a priori. These a priori color choices did not exert any influence on the methods of analysis or results.

A visual inspection of the distribution of the clusters in Fig 1 shows the following stylized features: (1) Health Sciences and Mathematics tend to share different clusters than those of Social Sciences; (2) the network structure shows several consistent patterns over time for both low-income and high-income countries; (3) Business and Economics are very close in half of the clusters, but they seem to become more isolated from other disciplines more recently in high-income countries; (4) Engineering, Physics, and other STEM-related sciences are often clustered or are related through inter-cluster links, demonstrating their commonalities; (5) for low-income countries, the associations between Agricultural, Veterinary, and Immunology are strong and consistent; and (6) Mathematics and Computer Sciences form a strong inter- or intra-cluster link in all but one sub-period and income cluster, forming one of the most consistent linkages among all pairs of disciplines.

While most of the same features remain across income levels, the networks appear to show greater dynamics in the middle-income clusters.

Fig 2 allows for analysis of the relative productivity of the different countries. Color-coding was used solely for visual representation of research disciplines and countries considered similar a priori, and was not used in the actual analysis (the expected outcome being that disciplines or countries with similarities would be linked), except when performing cluster-specific analysis by country income classification. Black edges represent intra-cluster linkages, while red edges represent inter-cluster linkages.

These graphs unveil that different income countries differ in the structure of the relative importance of research disciplines. The large number of high-income countries (yellow circles) linked either through intra-cluster or inter-cluster edges, indicates that they are not only intertwined economically, but also in terms of their research productivity profiles. This over-representation of high-income countries also indicates that the relative distribution across disciplines of the research productivity can serve as a predictor of the economic status of the country. Since these results are normalized by total research productivity, the network inputs are not dependent on the size or availability of resources dedicated to research in the country, and only conditioned on how those resources are distributed and the corresponding relative outcomes in research productivity across disciplines.

**Fig 3:**
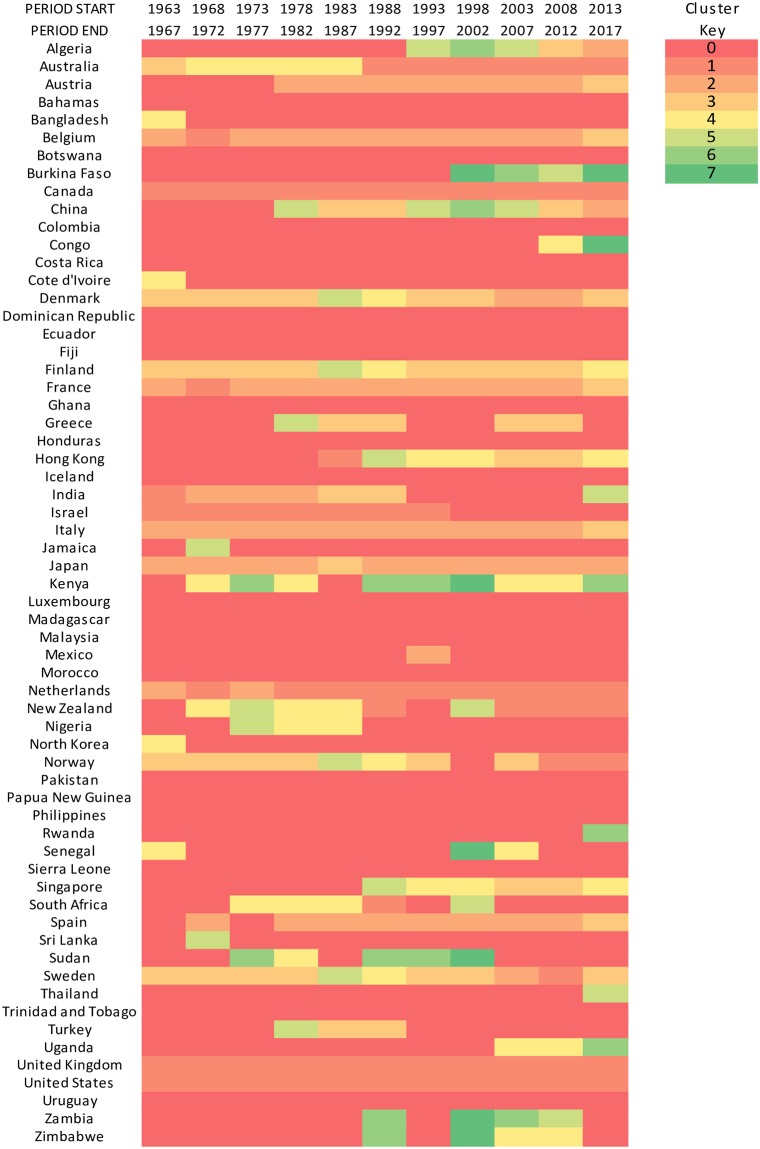
Clusters of research relative weights to which countries belong during different periods in time. Membership to a cluster does not indicate importance of the cluster or the country's research, but instead similarities in relative productivity across disciplines for those countries. Cluster identifiers do not follow any particular ordering.

Fig 3 displays results of the cluster analysis of different countries, grouped according to the similarities of their research systems and processes, which are assessed by the distribution of research disciplines according to their research productivity in each country. Cluster 0 represents countries which do not have strong productivity research profile similarities with any other country. This does not mean that their research is not connected, but instead that the productivity research profile distribution across disciplines is more heterogeneous than that of the countries that appear in non-zero clusters. The majority of countries belong to this ‘zero’ cluster since higher similarities are found among higher income countries. There seems to be an Anglophone cluster (cluster 1), where some countries with strong ties to Anglophone countries (e.g., Netherlands and Norway more recently) are grouped in the research profile defined by the cluster of English-speaking countries (e.g., Canada, US, UK, Australia, New Zealand). Japan has closer ties to research profiles of Central and Southern Europe (in most periods this is represented by cluster 2) than to that of Anglophone countries.

There is strong cluster stability over time: countries rarely move from one cluster to another. On average, countries remain in the same cluster as the previous period in approximately 75% of cases. Spain consistently joined the Mediterranean cluster in the late 70s, when democracy was introduced, there were large investments in education, and stronger links to Europe were politically sought. Despite the geographical proximity, Netherlands and Belgium have markedly different ties, with the latter belonging more to the central-to-southern European cluster. Geopolitical ties matter: for example, Hong Kong and Singapore have very similar paths of cluster membership. Similar features are found more recently for Uganda and Kenya. Glimpses of a commonwealth effect can be seen between South Africa and New Zealand, though those have been less relevant since the turn of the century. Among larger economies, China shows greater variability in cluster membership, probably a feature of its larger-than-average economic dynamics over time, as well as their historically extreme contrasts between the times of the Cultural Revolution and the more recent era of scientific advancements. Interestingly, China and Japan, which share a history of conflict, have more recently (2013–2017) developed a strong level of similarity in their research profiles, which coincides with larger commercial exchanges between the two countries. Algeria’s link to China could be spurious, though Algeria shares strong commercial ties with China, Algeria’s largest supplier by a large margin, and has maintained strong historical relations. Israel used to belong to the Anglophone cluster, but that relationship broke at the turn of the century.

Table 3 provides a ranking of each discipline by income cluster. This ranking identifies, for each income cluster, the disciplines with larger relative share of research publications compared to other income clusters. For example, Psychology has the largest relative share of publications within high-income countries compared to the other income clusters for that discipline, while Arts has the second largest relative share of research publications among high-income countries versus the rest of the clusters for that discipline. Veterinary, Immunology, Agriculture, and Medicine are disciplines where low-income countries have a larger relative share, which aligns with their needs. Similarly for high-income, advanced economies, leisure and health (mental and physical) account for the top five disciplines. Again, these rankings are not built in terms of absolute numbers of publications, but relative proportions as a percentage of the total research compared to other income clusters. Therefore, they are not influenced by total research productivity or country/cluster size.

**Table 3:** Ranking of relative shares of discipline productivity grouped by income cluster (not actual research publications, but discipline publication shares as a percentage of the total within the discipline) in 2017.

	Low	Low-medium	High-medium	High
1	Veterinary	Pharmacology, Toxicology, and Pharmaceutics	Material Science	Psychology
2	Immunology and Microbiology	Dentistry	Chemical Engineering	Arts and Humanities
3	Agricultural and Biological Sciences	Computer Science	Engineering	Nursing
4	Medicine	Veterinary	Chemistry	Health Professions
5	Environmental Science	Energy	Energy	Neuroscience
6	Social Sciences	Engineering	Mathematics	Social Sciences
7	Economics, Econometrics, and Finance	Decision Sciences	Physics and Astronomy	Economics, Econometrics, and Finance

## Conclusions

### Discussion

The nature of scientific contributions in the form of published research offers a window into the relationships among the disciplines, as well as their association with the economic status of a country. Among some of the key features unveiled in this manuscript are: (1) countries with high (per capita) income have similar structures of their research productivity in relative terms, forming a strong network with identifiable geographical, linguistic, commercial, and geopolitical links; (2) some disciplines have strong relationships that remain across all country income levels (such as Computer Sciences and Mathematics), while others are more dependent on the income level of the country and their reliance on the primary sector (Agriculture and Veterinary); (3) China appears more recently within the network with similar research productivity profiles to high-income Asian countries, though it shows a history of swings that aligns with an erratic view of their research community; (4) countries outside the high-income group tend to form small isolated clusters mostly driven by geographical proximity, rather than income status; (5) middle-income countries show more dynamics in the discipline network than countries in lower and higher income brackets. This could be due to either funding deficiencies hindering dynamics (low income countries) or consolidation of relative importance of research disciplines for high-income countries and rigidities to modify the status quo (e.g., politics around budgets and departmental expansions); and (6) disciplines such as Psychology and Arts are over-weighted (in relative terms) in high-income countries, while disciplines such as Agriculture and Immunology are more relevant and remain over-weighted for low-income countries. This is reflective of the different concerns that wealthier countries face (mental health, leisure) versus those of lower income countries (food security, survival) [28]. Diminishing interdisciplinary relations, such as those mentioned regarding Economics and Business, can be concerning given the need to tackle ever more complex problems [29].

Higher dynamics in middle-income clusters could be due to those countries exposed to less pressure to maintain the status quo than other income clusters. Low-income clusters could be more bounded in their evolution by their constraints (scientific and financial) to shift resources between disciplines. High-income clusters could be more bounded in their evolution by structural constraints (e.g., inter-departmental budget fights and shared governance may induce a higher propensity to maintain the inter-disciplinary status quo).

The method used in this manuscript allows for identification of clusters of countries that may be considered ‘aspirational’ for developing countries for economic or geopolitical purposes, allowing for identification of long-term distributions of resources among disciplines to match those in targeted clusters. The present work did not assume any clusters of scientific fields a priori, but identified the clusters based on the data. This is different from studies which assume a priori a particular clustering of disciplines [30], which may explain why those studies found that national wealth was not associated with tradeoffs between altruistic and economic motives. While finer methods for clustering areas of research based on publication data have been performed [31,32], the work presented in this manuscript focused on a more course approach to extract more robust relationships and their dynamics over time.

This study does not engage in causality arguments. The causal relationship between research productivity and economic advancement has largely been discussed in the literature [6–11]. On that basis, this study provides a benchmark for developing countries to define their research ecosystem, as a means to pursue economic advancement. Hence, this study broadens the window on the dynamics of the research ecosystem among countries. It helps pinpoint critical aspects of this ecosystem in each country and helps to plan for future developments.

### Study limitations and future research

The scope of this study is bounded by the number of countries for which the data was: (1) complete and reliable across years and disciplines; (2) not affected by country expansions/territorial redefinitions; and (3) available from the World Bank with regards to GDP per capita country classification. This led to a universe comprised of 62 countries with complete history, and has the potential to affect some regions more than others. While the study largely expands work done in this area, it is not comprehensive and does not cover all countries throughout the world. Countries that experienced major geographical integration or disintegration processes (e.g., Germany or Russia) were not included, to avoid artificial jumps mostly driven by geopolitical events rather than scientific dynamics. However, while partial in nature, this study expands the literature on relationships among research disciplines and their relationship with economic clusters over time.

Expanded or sub-group analyses are feasible for a larger list of countries if constrained solely to more recent periods. This would allow for geographical or geopolitical sub-analyses. For example, data for all countries in South America would be available for more recent years, and a network comprising only these countries could be constructed to explore in more depth the intra-region relationships. Such networks would be less affected by the strength of the similarities found within higher income countries, which dominate the analyses in this manuscript. However, while possible through the methodology introduced in this manuscript, this type of more granular analysis is out of the scope of this study.

Throughout this manuscript, a link between country wealth and research profile is explored. When those associations exist, causation cannot be claimed within the scope of this manuscript. Also, there is no evidence that those associations are not driven by a set of other common/latent factors (e.g., political factors, geographical factors, individualism, level of penetration of capitalism, access to national/transnational public/private funding of research, perceptions regarding the relevance of higher education, etc.).

Finally, the mapping of the research literature to countries or disciplines is arbitrary and does not follow any validated or commonly-agreed structure. There are multiple ways in which each piece of research can be assigned to countries and disciplines, all of which would show different angles to the same questions. A comparison of all those forms, while interesting, remains outside the scope of this manuscript.

## Supporting information

S1 Data (XLSX)
